# *Medicago sativa*’s antixenotic and antibiotic resistance mechanisms differentially impact three members of the *Bemisia tabaci* species complex

**DOI:** 10.1038/s41598-025-01426-z

**Published:** 2025-10-16

**Authors:** Patrick Thomas, Mohamed Ali Benabderrahim, Jun Li, Min Jiu, Lunji Wang, Frances Holzer, Larry Teuber, Linda L. Walling

**Affiliations:** 1 Department of Botany and Plant Sciences, University of California, Riverside, CA 92521 USA; 2 Faculty of Sciences of Tunis, Department of Arid and Oases Cropping Laboratory, University of Tunis El Manar, Institut des Régions Arides, Medenine, 4119 Tunisia; 3 Department of Statistics, University of California, Riverside, CA 92521 USA; 4 College of Food and Bioengineering, Henan University of Science and Technology, Luoyang, China; 5 Key Laboratory of Microbial Resources Development and Utilization, College of Food and Bioengineering, Henan University of Science and Technology, Luoyang, China; 6 Department of Plant Sciences, University of California, Davis, CA 95616 USA; 7 Institute of Integrative Genome Biology, University of California, Riverside, CA 92521 USA; 8 Present Address: Department of Plant Sciences, Pennsylvania State University, University Park, PA 16801 USA

**Keywords:** Antibiosis, Antixenosis, *Bemisia tabaci* (whitefly), Insect performance, *Medicago sativa* L. (alfalfa), Host plant resistance, Plant sciences, Plant stress responses, Herbivory

## Abstract

The *Bemisia tabaci* species complex threatens worldwide agriculture. While host-plant resistance is sustainable and effective, it is a relatively unexplored strategy for whitefly control. Three alfalfa (*Medicago sativa* L.) populations were developed and used in high-throughput *B. tabaci* MEAM1 nymph-mortality screens. The phenotypic resistance/susceptibility spectrum in these populations indicated that whitefly resistance to MEAM1 is multigenic. Alfalfa lines highly resistant (R1, R2, and R3) and susceptible (S1) to *B. tabaci* MEAM1 were identified and further characterized. When life history parameters for three *B. tabaci* species (MEAM1, MED and NW1) were assessed on the four lines, whitefly species-specific responses to R1, R2 and R3 plants were revealed. While nymph mortality was high for MEAM1 and NW1 on resistant alfalfa, MED nymph development was surprisingly unimpaired. In addition, significant differences in oviposition, host-choice and longevity were observed amongst the three *B. tabaci* species. Collectively, these data indicated that R1, R2 and R3 plants each express a unique set of antibiotic/antixenotic resistance traits providing a potent multigenic and multi-faceted resistance to whiteflies with each *B. tabaci* species displaying distinct behaviors on the resistant lines. Our nymph-mortality simulation models indicate that deployment of the nymph mortality-mediated resistance of R1 and R3 alfalfa could substantially suppress whitefly population expansion in the field.

**Supplementary Information:**

The online version contains supplementary material available at 10.1038/s41598-025-01426-z.

## Introduction

Two-thirds of hemipteran insects with sequenced genomes are classified as “high-status” pests that cause massive devastation in protected and field agriculture^2^. Among these pests, whiteflies of the 40-member *Bemisia tabaci* cryptic species complex are some of the most omnipresent and invasive pests worldwide, with at least one *B. tabaci* species extant on every continent except Antarctica^3,4^. *B. tabaci’s* voracious feeding inhibits plant growth and development due to depletion of resources, enables virus transmission, and promotes honeydew secretions that support sooty mold growth on plant surfaces^5–7^. Whiteflies are difficult to control due to abaxial oviposition, ability to rapidly develop insecticide resistance, ability to avoid effective defenses, and, with a few notable exceptions, limited success in deploying natural enemies in agricultural field settings^8–11^.

*Bemisia tabaci* MEAM1 (Middle East Asia Minor 1) is recognized for its global pest status^12^. In the early 1990’s, MEAM1 invaded the American southwest and rapidly replaced the native *B. tabaci* NW1 (New World 1)^13^. Coupled with its larger host range, ability to vector a wider range of viruses, and greater fecundity, the invasive MEAM1 remains a larger threat to agricultural systems than its native counterpart^6,13–15^. In the US, superabundant MEAM1 populations have been associated with numerous crops including *Solanum lycopersicum* (tomatoes), (*Lactuca sativa*) lettuce, (*Brassica oleracea*) cauliflower, (*Cucumis melo*) melon, (*Gossypium hirsutum*) cotton, (*Cucurbita pepo*) squash, and (*Medicago sativa*) alfalfa reducing yields, crop quality and promoting whitefly intercrop movement^16^. In addition, MEAM1 causes plant physiological disorders that significantly decrease the value of crops including leaf silvering of squash, irregular ripening of tomato fruit and stem blanching in *Brassica*^17–22^. The invasive *B. tabaci* MED (Mediterranean) is well established in Europe and China; while MED has yet to spread into the agricultural field settings in the US, it is present in greenhouses and nurseries^23,24^. Both MEAM1 and MED displaced native *B. tabaci* species in China and MED’s propensity for developing insecticide resistance has led to its growing impact in China^25,26^. The prevalence of whiteflies and their widespread damage globally have heightened the urgency for alternative control methods.

Host-plant resistance is foundational for integrated pest management programs^8,27,28^. Surprisingly, relatively few genes that confer resistance to hemipteran pests have been successfully cloned and characterized. These genes include nine rice genes conferring resistance to brown planthopper (*Nilaparvata lugens*) (*Bph2/26*,* 3*,* 6*,* 9*,* 14*,* 17*,* 18*,* 29*,* 32*), the tomato *Mi-1.2* that confers resistance to three hemipteran pests, the melon gene (*Vat*) that confers resistance to cotton-melon aphid (*Aphis gossyppi*), and the wheat gene (*Adnr1*) that confers resistance to the Russian wheat aphid (*Diuraphis noxia*)^29–34^. With two exceptions (*Bph3* and *Adnr1*), hemipteran resistance genes are coil-coiled nucleotide-binding leucine-rich repeat receptors (CC-NLRs).

*Mi-1.2* is a unique as it confers resistance to whiteflies (*B. tabaci* MEAM1 and MED), root-knot nematodes (*Meloidogyne spp.*), potato aphids (*Macrosiphum euphorbiae*), and psyllids (*Bactericerca cockerelli*)^35–39^. While *Mi1.2* resistance to aphids is phloem mediated, the resistance to whiteflies in apoplastic^40^. In addition, broad-spectrum trichome-dependent resistance to insects, including whiteflies, is present in wild tomato species^39,41–43^.

Resistance to other whitefly genera has been characterized in cabbage (*Brassica oleraceae)* and cassava (*Manihot esculenta).* A phloem-mediated resistance to *Aleyrodes proletella* (the cabbage whitefly) was discovered in *B. oleracea* and is associated with the phytohormone abscisic acid^44–46^. Cassava’s resistance to the Latin American whitefly *Aleurotrachelus socialis* is associated with lignification to reduce oviposition, prolong nymph development and increase nymph mortality^47,48^. Abscisic acid is positively correlated with cassava’s whitefly resistance^49^. Whitefly resistance has also been identified, but not extensively characterized in wild and cultivated cotton^50,51^, wild watermelon^52^ and a number of legumes (e.g., soybean, common bean, cowpea, mungbean, and black gram)^53–57^.

Alfalfa (*Medicago sativa* L.) has a novel, trichome-independent resistance mechanism to MEAM1^58–60^. At high whitefly densities, alfalfa suffers from decreases in plant growth, stem lengths, forage yields, dry matter production, and protein content^5^. In addition, as a high-value seed crop, alfalfa is often intercropped adjacent to other high-value crops making it a potential reservoir for whitefly expansions into agricultural operations^8^. A field screen of half-sib families from the genetically diverse UC-356 germplasm, which is used in alfalfa breeding, identified 73 MEAM1-resistant alfalfa plants and 73 half-sib families were made^60^. A shuttle breeding scheme in California and Chile was used to select for whitefly resistance^60^. This multi-year project identified alfalfa resistance to MEAM1 based on decreased numbers of nymphs and reduced honeydew production^60^. In 2003, 17 potentially resistant and 21 presumed susceptible alfalfa individuals were characterized for resistance/susceptibility in controlled environments in Jiang et al.^59^. Whitefly-resistant individuals have reduced phloem consumption by nymphs and increased nymph mortality^58^.

Despite its promise for whitefly control, alfalfa’s complex genetic composition and unique breeding strategies makes genomic analyses challenging^61–63^, which has resulted in little progress in elucidating the function and identity of alfalfa’s whitefly-resistance mechanism. Here, we provide a foundation for better understanding alfalfa’s resistance to whiteflies. Three alfalfa populations derived from plants from UC-356 germplasm were made. Eighty-four alfalfa lines from these populations were screened for whitefly resistance using MEAM1 nymph mortality as the resistance indicator. Three highly resistant lines and one susceptible line were used to explore the impact of these whitefly-resistance mechanisms on four whitefly life-history parameters (oviposition, nymph development, adult longevity, and adult preference) of three members of the *B. tabaci* species complex (MEAM1, NW1 and MED).

## Results

### Identification of MEAM1-resistant alfalfa

**Fig. 1:**
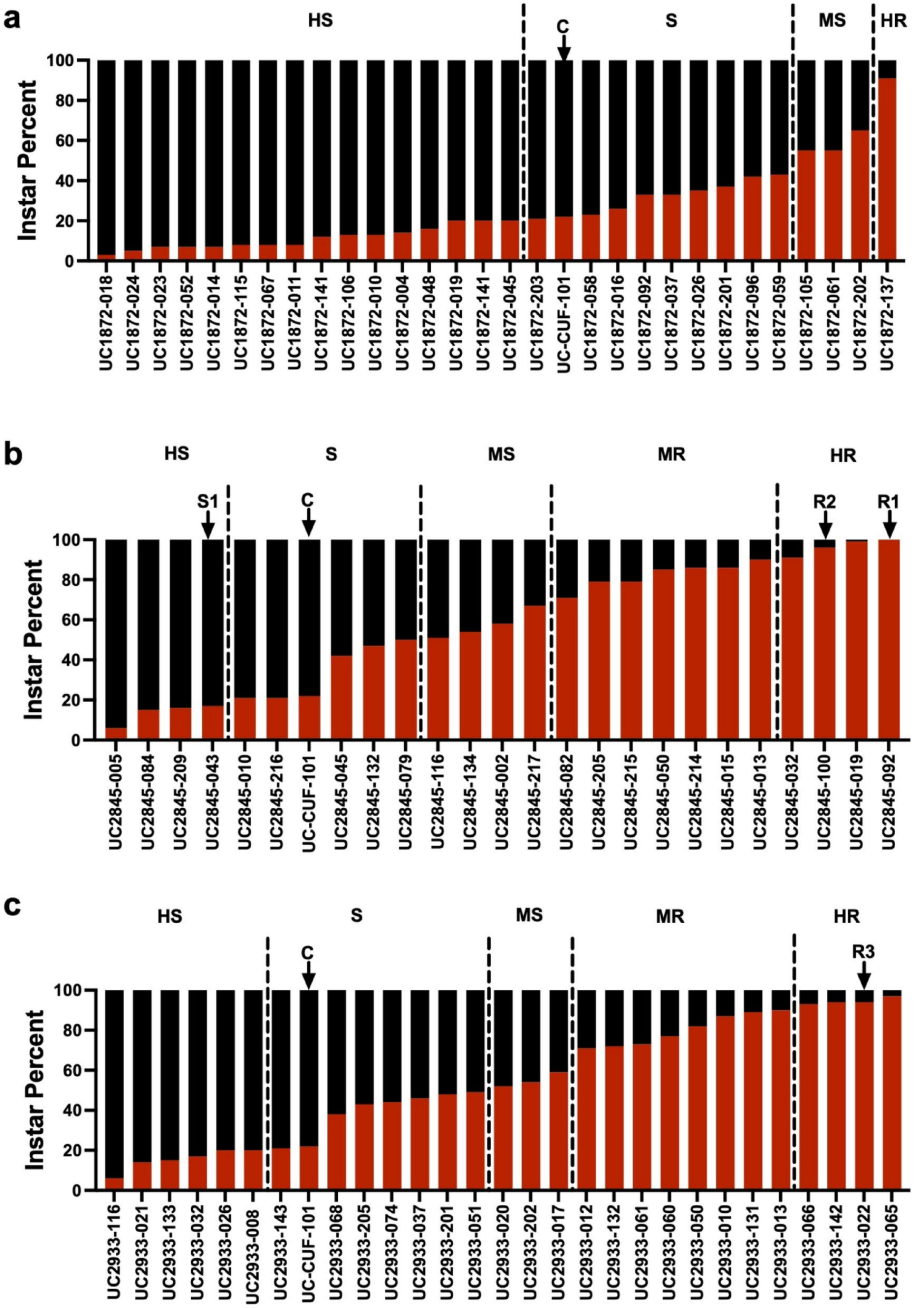
*B. tabaci* MEAM1 nymph development on individuals from the UC-1872, UC-2845 and UC-2933 populations. The percentage of insects in their first instar (red) vs. later instars was determined for 29 UC-1872 lines (**a**), 25 UC-2845 lines (**b**), and 28 UC-2933 lines (**c**) and resistance classes were assigned. The positions of CUF-101 (C), S1, R1, R2, and R3 genotypes along the susceptibility-resistance spectrum in these screens are indicated. A representative screen is shown in Supplementary Figure S2 and complete data set is found in Supplementary Table S1.

One whitefly-susceptible population UC-1872 and two whitefly-resistant alfalfa populations UC-2845 and UC-2933 were created from the UC-356 germplasm (Supplementary Figure S1). From these populations, 84 lines were screened for resistance/susceptibility to *B. tabaci* MEAM1 using a block of nymph development as a scoring metric (Fig. 1). On the whitefly-susceptible CUF-101, ~ 78% of the nymphs progressed beyond the first instar; while on resistant plants, few insects developed beyond their first instar reflecting nymph mortality or a severe developmental delay (Fig. 1; Supplementary Figure S2). Plants were assigned to one of five resistance/susceptibility classes: highly resistant (> 90% first-instars), moderately resistant (> 70–90%), moderately susceptible (> 50–70%), susceptible (> 20–50%), and highly susceptible (0–20%) (Fig. 1, Supplementary Figure S3). Aligned with the methods used for alfalfa cultivar breeding and with resistance being conferred by multiple genes, each population had a spectrum of phenotypes ranging from highly susceptible to highly resistant (Fig. 1; Supplementary Figure S3)^60^.

Correlated with the selection of UC-1872 for whitefly susceptibility, 97% of UC-1872 plants were moderately to highly susceptible to MEAM1 (Fig. 1a, Supplementary Figure S3). Seventeen UC-1872 lines were as or more susceptible than the known whitefly susceptible CUF-101^59^. Consistent with the strategies for alfalfa breeding, which preserve and promote genetic diversity in breeding populations, one highly resistant plant (UC-1872-137) was identified in the UC-1872 population (Fig. 1a). The two whitefly-resistant populations (UC-2845 and UC-2933) had significantly more lines exhibiting resistance, with 46% and 43% of the lines exhibiting moderate to high resistance, respectively (Figs. 1b-c; Supplementary Figure S3). Although the UC2845 and UC2933 populations were created using different breeding strategies, there was no significant difference in the number of either moderately and highly resistant plants (*p* > 0.99) or highly resistant plants (*p* > 0.99) in the two populations (Supplementary Figure S3).

 Three lines UC2845-092 (R1), UC2845-100 (R2) and UC2933-022 (R3) expressing a strong antibiosis that blocked MEAM1 nymph development and one highly susceptible line UC2845-043 (S1) were chosen for further evaluation (Fig. 1). R1 and R2 displayed a robust resistance with 99% and 96% of MEAM1 nymphs remaining in their first instar, respectively. Line R3 was the second-most resistant line in the UC2933 population with 94% first-instar nymphs.

**Fig. 2:**
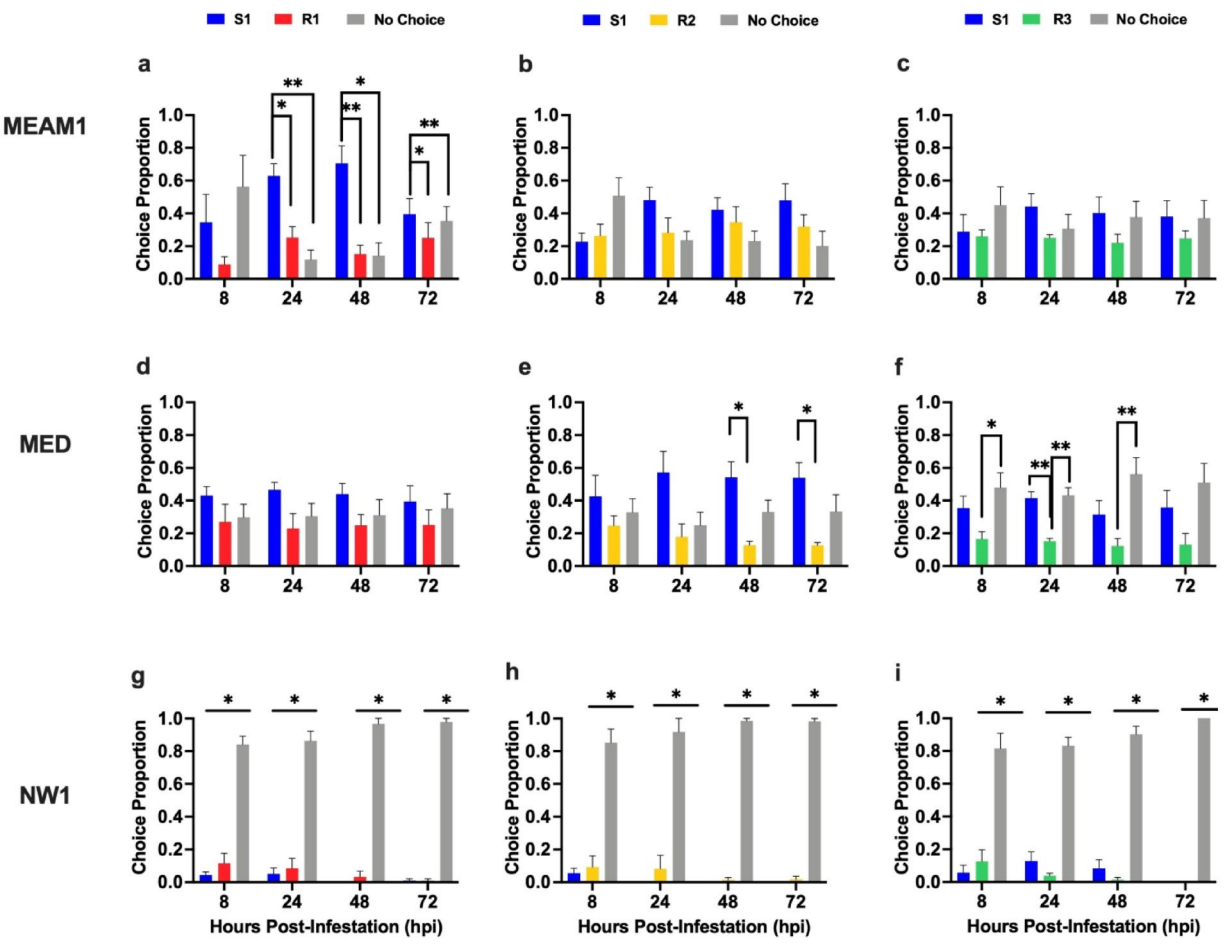
Free-choice experiments. MEAM1 (**a-c**), MED (**d-f)** and NW1 (**g-i**) choice studies. The proportion of *B. tabaci* adults that chose S1 or a resistant genotype R1 (a, d, g), R2 (b, e, h) or R3 (c, f, i) or made no choice is shown. Statistically significant differences are marked with asterisks (* = 0.05, ** = 0.01, *** = 0.001).

### MEAM1 and MED adults are not attracted to whitefly-resistant alfalfa

To determine if R1, R2, or R3 express active antixenotic mechanisms, choice assays were performed to assess MEAM1, MED, or NW1 adult settling on S1 versus a resistant (R1, R2 or R3) plant (Fig. 2; Supplementary Table S2). For MEAM1, there was a strong preference for S1 over R1 plants at all times (Fig. 2a). In contrast, MEAM1 did not discriminate between S1 and R2 nor S1 and R3 (Figs. 2b-c); however, there are trends that suggested S1 was preferred over R2 and R3 at later times (24–72 hpi). Unlike MEAM1, MED did not discriminate between S1 and R1 plants at any time point (Fig. 2d) and MED preferred S1 over R2 plants (Fig. 2e). In the S1/R3 choice experiments, MED preferred S1 over R3 plants at 24 hpi (Fig. 2f); in addition, a higher percentage of MED adults did not make a choice in these bioassays.

NW1 displayed a dramatically different interaction with S1 and the three resistant hosts. NW1 did not discriminate between the susceptible and resistant genotypes in the S1/R1, S1/R2, or S1/R3 two-choice assays. Instead, over 80% of NW1 adults avoided settling on any alfalfa plant (Figs. 2g-i). All NW1 adults died at the conclusion of each experiment, likely from starvation.

**Fig. 3:**
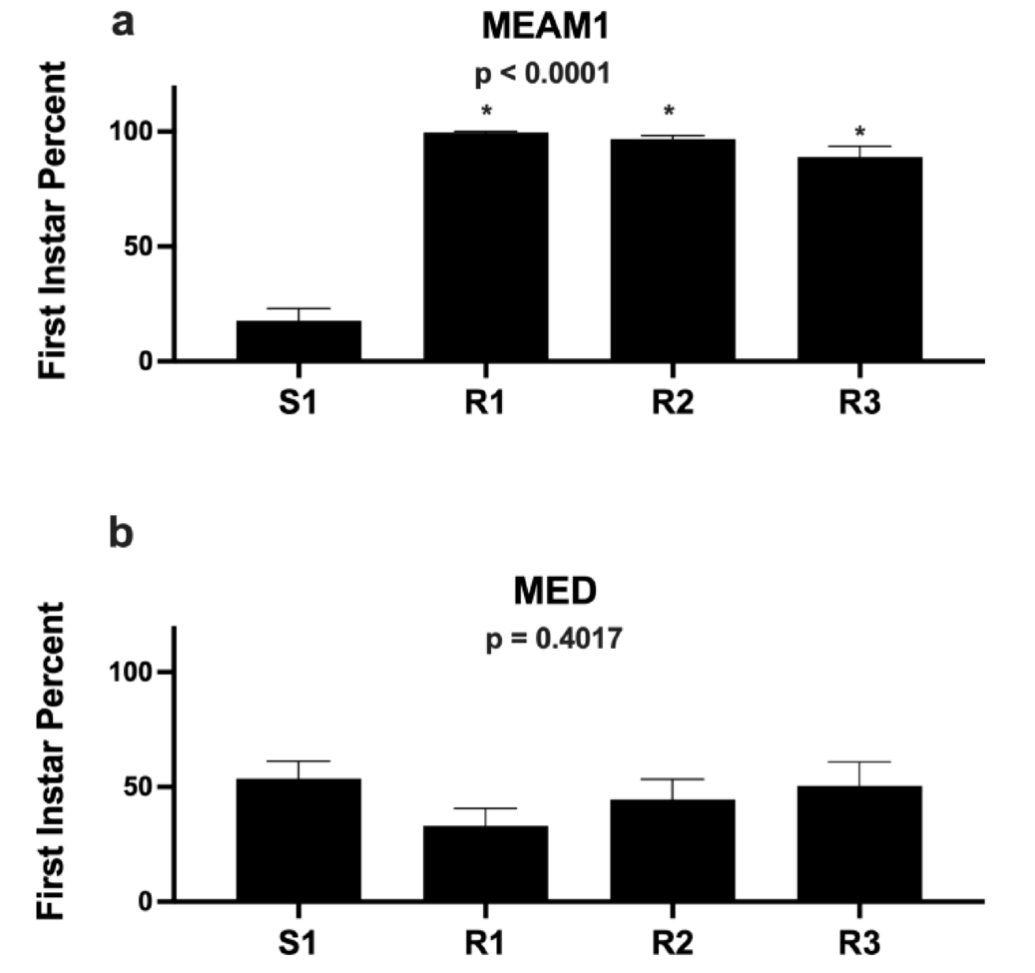
The MEAM1 and MED nymph development on whitefly-susceptible and -resistant alfalfa The percentage of first-instar MEAM1 (a) and MED (b) nymphs on S1, R1, R2 and R3 plants. Each experiment was compared using a Kruskal-Wallis one-way ANOVA on arcsin square root of the proportion of first-instars of each replicate. *p* values are shown.

### MED nymphs develop on the R1, R2 and R3 alfalfa lines

We evaluated if the antibiotic resistance that caused MEAM1 nymph mortality in resistant alfalfa plants impacts MED and NW1 nymph development. As seen in the large-scale phenotypic screens (Fig. 1), MEAM1 nymph development was significantly delayed on the three resistant genotypes relative to S1. While 83% of MEAM1 nymphs progressed beyond the first instar on S1, *≤* 6% of the nymphs progressed beyond their first instar on the resistant plants (Fig. 3a). In contrast, MED nymphs were able to develop at similar rates on all four genotypes (*p* = 0.40) (Fig. 3b). When comparing MED and MEAM1 nymph development on S1 plants, MED developed more slowly, which might indicate that alfalfa was a less suitable host for MED.

**Table 1:** Development of NW1 whiteflies on susceptible and resistant alfalfa.

Alfalfa genotype	Whitefly-resistance class^a^	Percentage of whiteflies in each developmental stage					
		First Instar	Second Instar	Third Instar	Fourth Instar	Exuvia	Total Nymphs
CUF-101	S	49.02	25.49	21.57	3.92	0.00	51
UC-2845-050	MR	100.00	0.00	0.00	0.00	0.00	21
UC-2845-100 (R2)	HR	84.62	15.38	0.00	0.00	0.00	39
UC-2933-010	MR	57.32	25.61	17.07	0.00	0.00	82
UC-2933-022 (R3)	HR	94.12	5.88	0.00	0.00	0.00	17

^a^Whitefly-resistance classes are based on MEAM1 development (Fig. 1).

Given NW1’s extreme repellence of and adult mortality on R1, R2 and R3 (Fig. 2), we assessed NW1 nymph development on four whitefly-resistant alfalfa lines and the susceptible CUF101 (Table 1). Two lines were considered highly resistant (R2 and R3) and two moderately resistant (UC2845-050 and UC2933-010) based on the high-throughput screens that monitored MEAM1 nymph development (Fig. 1). Similar to MED1, NW1 nymph development was slower on the whitefly-susceptible host relative to MEAM1, suggesting alfalfa was a less suitable host for NW1. Given our small sample size, only two interactions were statistically significant and strong trends for other comparisons were evident. We found there was no significant difference in the proportion of NW1 first- (*p* = 0.076), second- (*p* = 0.094), third- (*p* = 0.12), and fourth-instars (*p* = 0.23) between genotypes. Notably, NW1 nymph development was completely blocked on the moderately-resistant line UC2845-050. Our analysis indicated that there were significantly more first-instars (*p* = 0.035) and fewer second-instars (*p* = 0.0275) on UC2845-050 than CUF-101. and strongly blocked on R2 and R3 plants. Rather surprisingly, on UC2933-010, which was moderately resistant to MEAM1, approximately 17% of the NW1 nymphs were able to develop into their third-star.

**Fig. 4:**
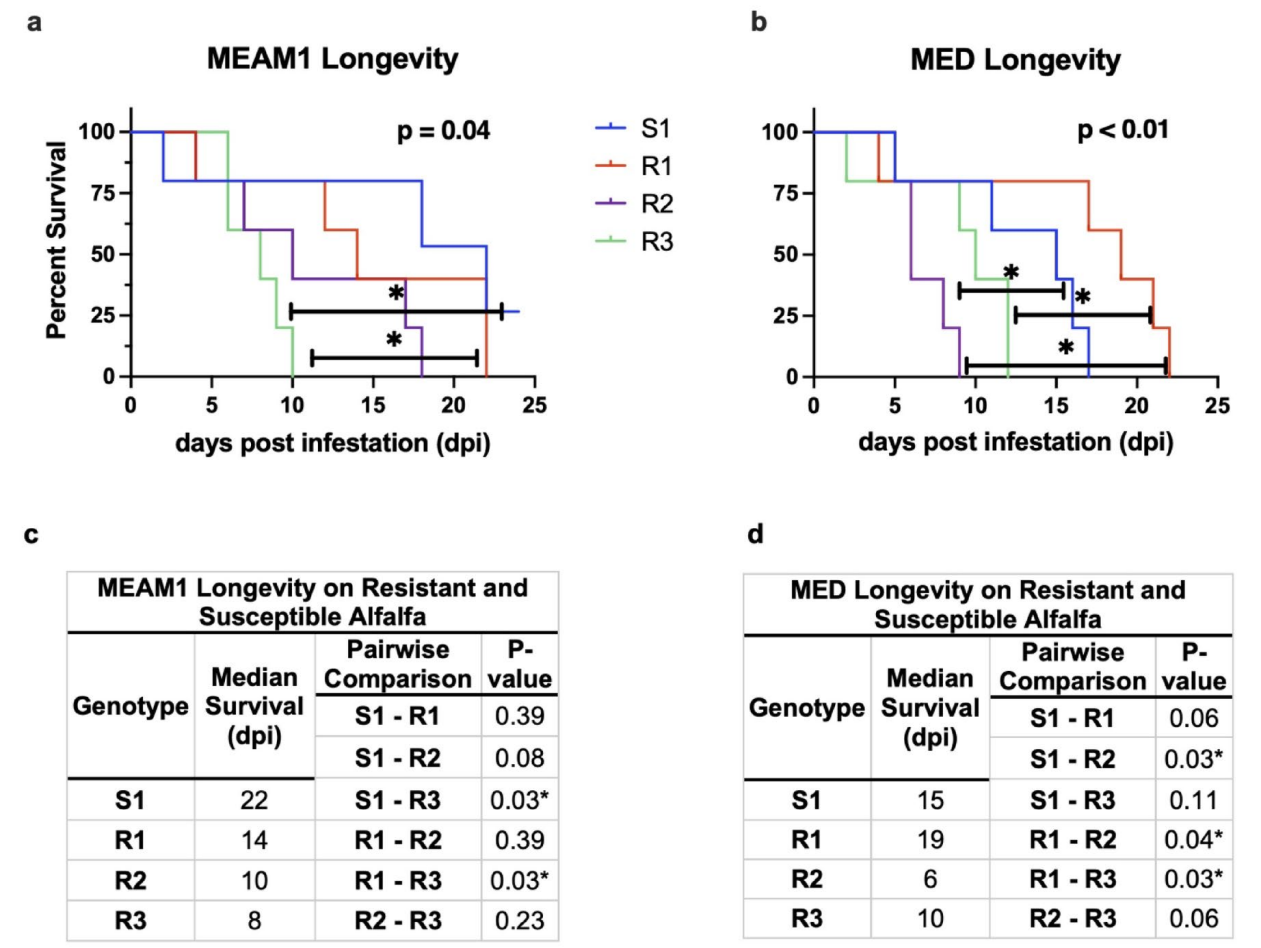
*B. tabaci* adult survival on S1, R1, R2, and R3 plants The viability of MEAM1 (**a**) and MED (**b**) adults on S1, R1, R2 and R3 alfalfa. Median survival of MEAM1 (**c**) and MED (**d**) adults. Pairwise comparisons were completed with a Mantel-Cox test of each survival curve. Significantly different values at the 0.05 confidence interval are indicated with an asterisk (*).

### Alfalfa’s resistance mechanisms impact adult longevity

We determined if the life span of MEAM1 and MED adults was influenced while feeding on S1, R1, R2, and R3. The three whitefly-resistant genotypes significantly influenced both MEAM1 (*p* = 0.04) and MED (*p* < 0.01) longevity, but in whitefly species-specific ways. For MEAM1, the adult lifespan was significantly shorter on R3 (8 d) relative to S1 (22 d) (*p* = 0.03) and R1 plants (14 d) (*p* = 0.03) (Fig. 4a and c). When comparing MED adult longevity on the four genotypes, we found a significant difference in the lifespan of S1 (15 d) and R2 (6 d) (*p* = 0.02) (Fig. 4b: d). Surprisingly, there was also a compelling trend for enhanced MED adult survival on R1 vs. S1. When comparing the lifespans of MED on the resistant lines, significant differences between R1 (19 d) and R2 (6 d) (*p* = 0.04) and R3 (10 d) (*p* = 0.03) were found. These experiments were attempted multiple times with NW1. However, consistent with the two-choice experiments, NW1 adults died 3 days after infestation in these no-choice experiments.

**Fig. 5:**
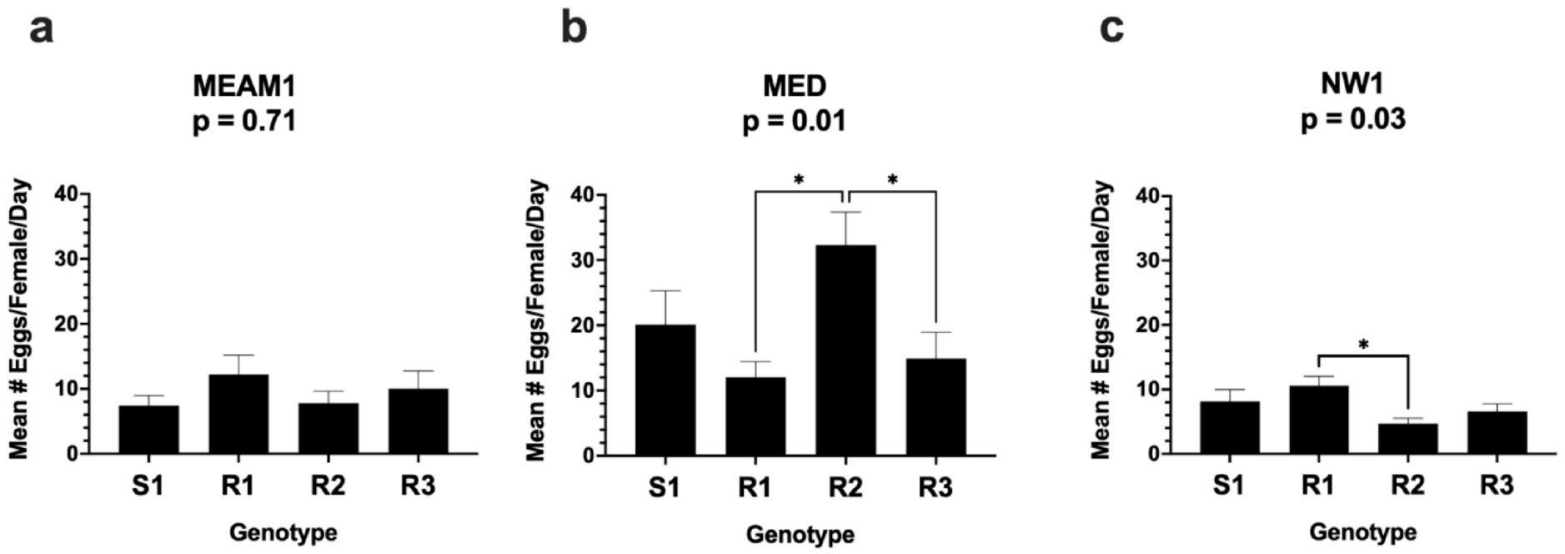
*B. tabaci* oviposition on S1, R1, R2, and R3 alfalfa The mean number of eggs/trifoliate leaf for MEAM 1 (**a**), MED (**b**), and NW1 (**c**) after 48 h was determined. Statistical significance of the means was assessed using a Kruskal-Wallis H test; p values appear in each panel. Statistically significant means within the MED and NW1 data sets were determined with a Dunn’s multiple comparison test at the 0.05 confidence interval are marked with an asterisk (*).

### MED and NW1, but not MEAM1, oviposition is influenced by alfalfa’s resistance mechanisms

We assessed if the R1-, R2- or R3-mediated resistance influenced MEAM1, MED, or NW1 oviposition (Figs. 5a-c). For MEAM1, MED and NW1, there was no significant difference in the number of eggs deposited on S1 vs. resistant genotypes. However, both MED and NW1 performed differently when the resistant genotypes were compared. For example, MED laid > 2-fold more eggs on R2 (32.3 eggs) than either R1 (12.1 eggs) or R3 (14.9 eggs) (*p* = 0.01 and *p* = 0.02, respectively). The impact of the R genotypes on NW1 oviposition was evident with > 2-fold NW1 eggs laid on R1 (10.6 eggs) than on R2 (4.7 eggs) plants (*p* = 0.03).

### The impacts of whitefly resistance on population growth – A simulation model

**Fig. 6:**
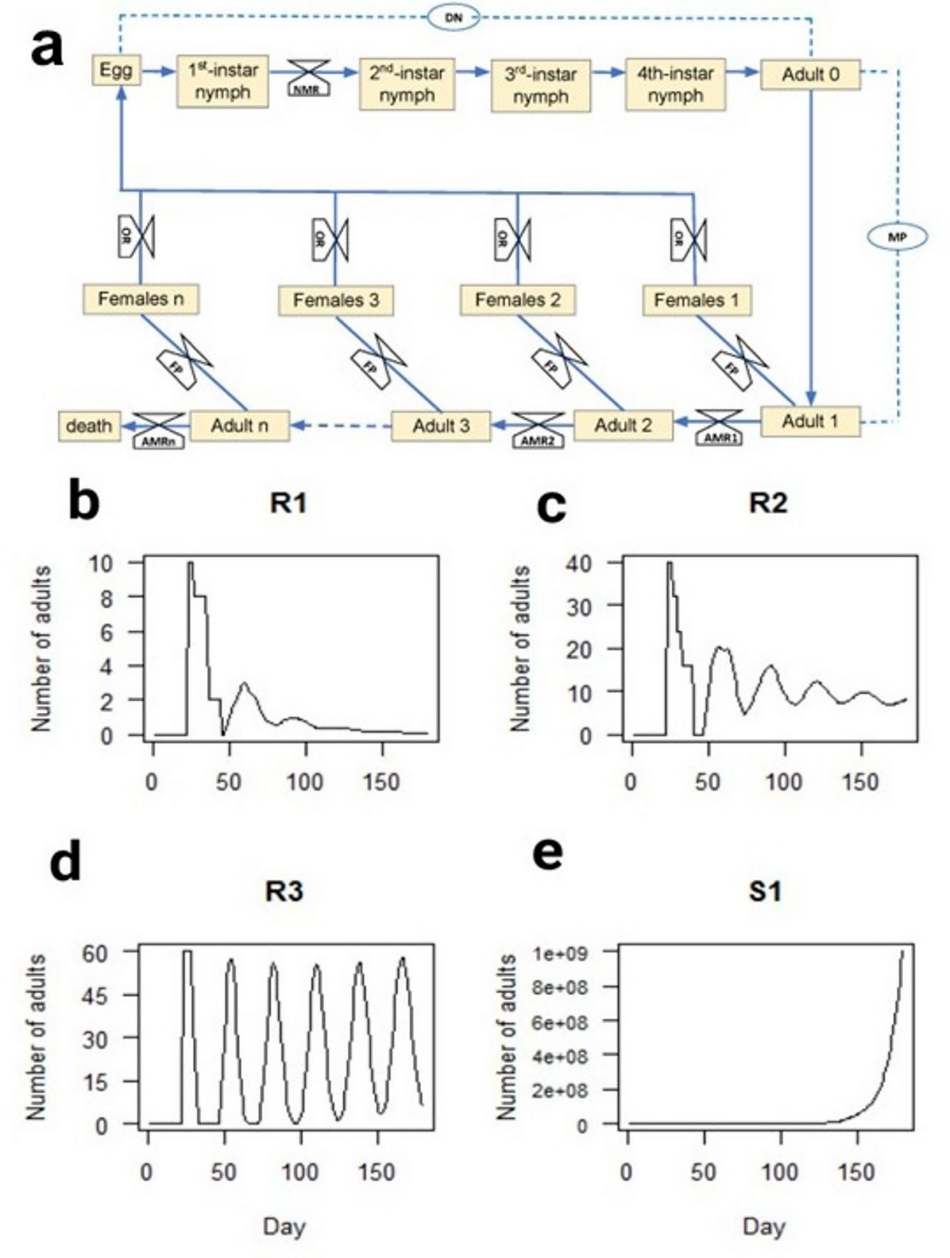
Simulation model for MEAM1 growth on whitefly-resistant and -susceptible alfalfa. (**a**) Simulation model scheme. Model parameters included: the duration of MEAM1 nymph development (DN = 23 days)^1^, female maturation period (MP = 1 day), female percentage (FP = 55%), nymph mortality rate (NMR) (Fig. 1), adult mortality rate (AMR, days) (Table 2) and oviposition rate (OR) (Fig. 5). (**b**) Population growth on R1. NMR = 99%, OR = 6.1, (**c**) Population growth on R2. NMR = 96%, OR = 3.9. (**d**) Population growth on R3. NMR = 94%, OR = 5.0. (**e**) Population growth on S1. NMR = 20%, OR = 3.

It has been proposed that 98–99% of nymphs must perish to effectively manage whitefly populations in the field^16^. To test if the resistance mechanisms deployed in R1, R2 and R3 plants meet this criterion, we developed a simulation model inspired by the model of Van Giessen et al.^64^ to predict how these whitefly-resistant alfalfa would impact whitefly population expansion. Figure 6a shows the flow chart describing various stages of whiteflies from eggs to adults used in our simulation model. Of the six parameters used in our MEAM1 model (Fig. 6a), three were alfalfa genotype-dependent including the nymph mortality rate (NMR) (Fig. 1), oviposition rate (OR) (Fig. 5), and adult mortality rate (AMR) (Fig. 4; Table 2). The remaining parameters were independent of the host-plant genotype: the duration of nymph development (DN)^1^; the female maturation period (MP); and percentage of females (FP) in a population. As there are various values for FP in the literature^65^, we used two FP values in our simulation models (FP = 55% and FP = 65%) (Fig. 6b, c, d, e; Supplementary Figure S4).

**Table 2:** MEAM1 adult mortality rates on whitefly-resistant and -susceptible alfalfa.

Alfalfa genotype	Number of days to achieve a mortality threshold				
	20%	40%	60%	80%	100%
R1	4 d	12 d	-	14 d	22 d
R2	4 d	7 d	10 d	17 d	18 d
R3	-	6 d	8 d	9 d	10 d
S1	2 d	12 d	18 d	22 d	24 d

Our model begins with 1000 eggs on an alfalfa plant (day 1). MEAM1 nymph mortality ranged from 20% (S1), 94% (R3), 96% (R2), and 99% (R1); mortality occurred during the first instar for all R plants. By day 23, the remaining viable nymphs have developed into adults with 55% (or 65%) of the adults being female. With a one-day delay prior to oviposition, the number of eggs laid is dependent on adult longevity (the AMR). We project population expansion over a period of 180 d (6 generations), which represents a typical 5-month growth period of alfalfa in California^1^.

Figure 6b-e show the number of MEAM1 adults during a 180-d period for R1, R2, R3, and S1 when FP = 55%. Cyclic bursts of population expansion (S1) or expansion and subsequent contraction (R1, R2 and R3) are predicted. On the whitefly-susceptible S1 line, there are few constraints to MEAM1 population growth and within six generations, over 1 billion adults are generated. R1 and R2 with NMRs of 99% and 96%, respectively, had negligible numbers of adults at 180 d, while R3 with an NMR of 94% had a very modest population growth reaching ~ 60 adults after 180 d. When FP was increased to 65%, the R1 and R2 population dynamics were unchanged (Supplementary Figure S4). In contrast, the R3 population increased but remained small (~ 120 adults). There were 2-fold more whiteflies predicted in the FP = 65% than the FP = 55% simulation. Similarly, S1 populations increased substantially, exceeding 2 billion adults in six generations.

**Fig. 7:**
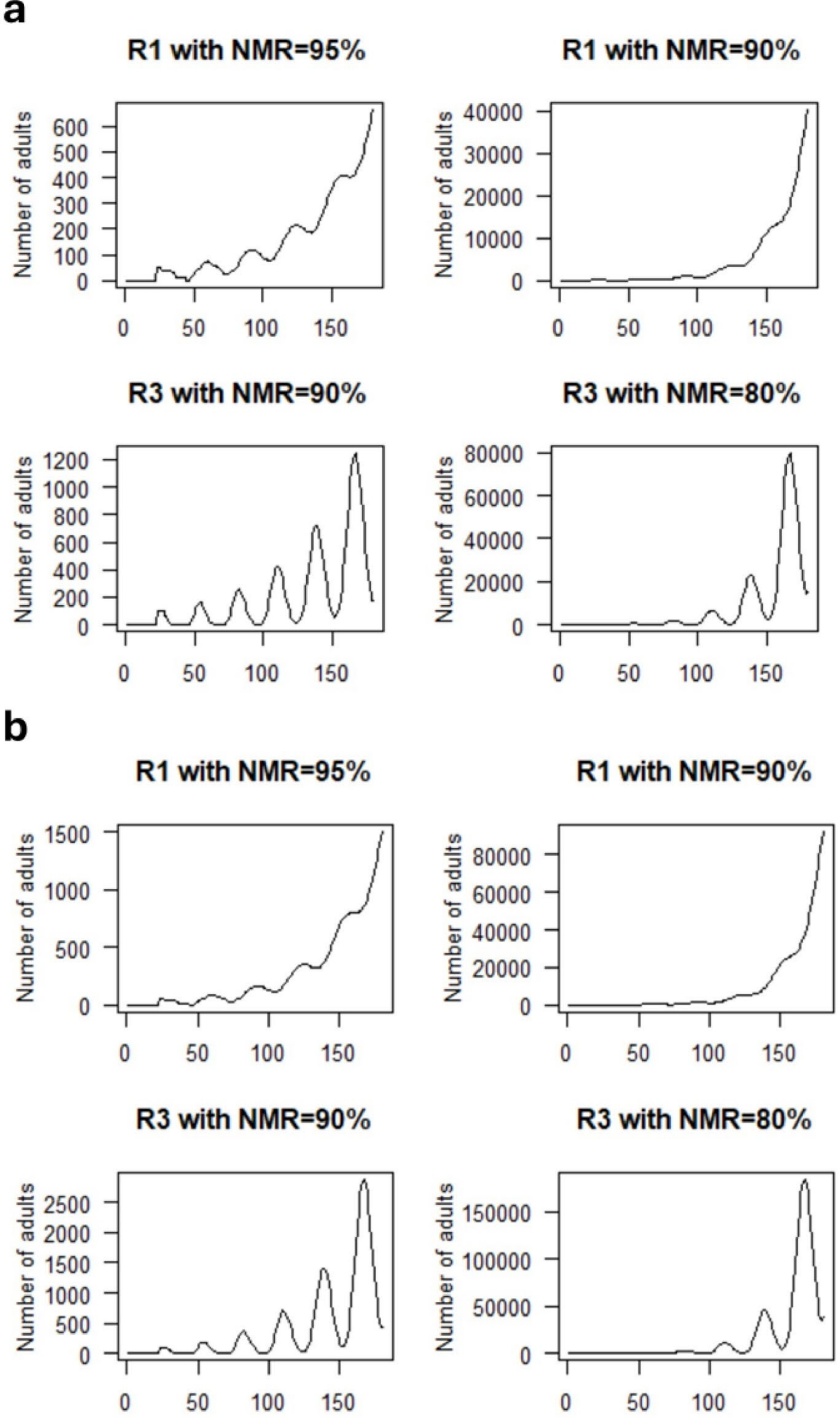
Simulation model for MEAM1 growth on R1 and R3 alfalfa with variable NMRs. The NMR and FP were varied to predict population growth thresholds in R1 and R3. The other parameters (DN, MP, OR, and AMR^20–80%^) used in the simulation model are the same as in Fig. 6. (**a**) Population growth on R1 and R3 with FP = 55%. (**b**) Population growth on R1 and R3 with FP = 65%.

Our model shows that NMR of 94% is sufficient to control whitefly populations over a 180-d period. This is likely due to the fact that longevity (AMR) and OR play critical roles in limiting population growth. As R1 and R3 had distinct NMRs (99% vs. 94%, respectively; Fig. 1), AMRs (22 d vs. 10 d, respectively; Table 2), and ORs (6.1 vs. 5.0 eggs/day, respectively; Fig. 5), we determined how a declining NMR would impact whitefly population dynamics (Fig. 7a). When the NMR for R1 was decreased to 95%, MEAM1 populations increased gradually over time, with ~ 600 adults at 180 d. With an NMR of 90% and the longer lifespan on R1, there was an exponential rise in the population reaching over 40,000 adults in 180 d. The kinetics for R3 population growth were distinct, as the R3 population continued to display oscillations across the 180-d simulation period when its NMR was lowered from 94 to 90% or 80%. While significant control was seen at 90% NMR for R3, at 80% NMR a scenario similar to the 90% NMR of R1 was seen. In both simulations, R3 populations were approximately 2-fold higher than R1. Increases in the FP to 65%, doubled the R1 and R3 population levels at all NMRs tested (Fig. 7b).

## Discussion

Given their voracious feeding habits, it is likely that the *B. tabaci* species complex will exert an increasing and substantial pressure on global agriculture in the future^66–68^. With the rise in global temperatures, *B. tabaci* populations are predicted to expand at unprecedented rates^67^. In response to elevated temperatures, the whitefly lifecycle may substantially accelerate and the growth seasons of whitefly host plants may lengthen; it is predicted that the number of *B. tabaci* generations in Mediterranean climates will increase from 9 to 12 generations annually, which will result in enormous increases in *B. tabaci* populations. Furthermore, increases in regional temperatures may expand habitats for the *B. tabaci* species complex into more northern latitudes causing new pressures on our agricultural systems. Given *B. tabaci*’s wide host range and ability to travel long distances on wind currents, *B. tabaci* will negatively impact the productivity and quality of high-value crops near its new habitats. As the largest acreage crop in California, mechanisms to curtail whitefly populations on alfalfa are needed. Due to fluctuating value of alfalfa hay, the high cost of insecticides, and the ability of haplodiploid insects (like whiteflies) to rapidly develop resistance to insecticides, chemical interventions are not always economically viable solutions for alfalfa^8,69^. For these reasons, there is an acute need to deploy alfalfa host-plant resistance.

To understand alfalfa’s mechanisms of whitefly resistance, high-throughput whitefly-resistance/susceptibility screens of three alfalfa populations were performed in a controlled environment. For the first time, we demonstrate that alfalfa’s resistance is a multigenic trait, as each population displayed spectrum of MEAM1-resistance phenotypes from highly susceptible to highly resistant. As there is a low probability of *B. tabaci* to alter its effector complement to overcome a multigenic resistance mechanism, the resistance revealed here has great potential for providing robust protection for numerous alfalfa generations. Three whitefly-resistant individuals (R1, R2 and R3) that displayed > 94–99% first-instar nymph mortality were selected for further studies. It is noteworthy that resistance phenotypes of our R1, R2 and R3 lines were similar to the whitefly-resistant alfalfa plants (clones 3, 10, 27, and 37) characterized in Jiang et al.^59^.

Modalities of plant resistance to pathogens/pests fall into three classes: antixenosis (non-preference of a host), antibiosis (the inhibition of development or survival of a pathogen/pest on a host), or tolerance (the ability of a host to limit symptoms of damage despite an active infection/infestation)^70,71^. Collectively, our assessment of nymph development, host choice, oviposition, and adult longevity suggest that both antibiotic and antixenotic mechanisms are active in MEAM1-resistant alfalfa. Quite surprisingly, the resistance mechanisms active in R1, R2 and R3 impact the three *B. tabaci* species in very different ways, supporting the premise that MEAM1, MED and NW1 are genetically distinct species with different adaptations to their host plants^4,72^.

Our whitefly life-history data indicated that R1, R2, and R3 alfalfa deploy resistance traits that differentially influence adult and nymph survival. For example, the antibiosis that caused MEAM1 and NW1 nymph mortality did not impact MED nymphs. In addition, the antibiotic mechanisms of the three resistant lines had different impacts on MEAM1 and MED adult longevity. The lifespans of MEAM1 and MED adults were shortest on R3 and R2 plants. While MEAM1’s lifespan was shorter on R1 vs. S1 plants; surprisingly, MED adults survived 4 d longer on R1 than on S1 plants.

Strong antixenotic resistance mechanisms are present in R1, R2 and R3, as both MEAM1 and MED preferred S1 over the resistant genotypes. The volatile and non-volatile phytochemicals that confer repellence in the R lines or attraction to S1 have yet to be determined and given the distinct *B. tabaci* species choices, the antixenotic mechanisms active in R1, R2 and R3 may be distinct. Furthermore, the decision of a female whitefly to deposit eggs on a host is partially a measure of host acceptance, as they first settle and then feed and oviposit concomitantly^73^. Surprisingly, the no-choice oviposition assays indicated that MEAM1, MED and NW1 deposited similar numbers of eggs on S1 and all three R genotypes; this suggests that the repellence mechanisms that deter adult colonization do not appear to influence oviposition under no-choice conditions. This contrasts with Jiang et al.^59^ who saw a weak correlation with MEAM1 nymph mortality and oviposition rates in the clonal lines that they analyzed. In addition, while MEAM1 did not discriminate between R1, R2 and R3, NW1 and MED discriminated between different resistant genotypes as oviposition hosts. NW1 preferred to oviposit on R1 plants and MED preferred R2 plants for oviposition. These data suggest that the differences in the antibiotic and/or antixenotic defenses deployed in R1, R2 and R3 alfalfa can be discerned by different members of the B. tabaci species complex.

In addition, the antibiotic/antixenotic resistance mechanisms expressed in R1, R2 and R3 lines are robust but appear to be mechanistically distinct. While all three R lines uniformly conferred an antibiosis to deter MEAM1 nymph development, it appears that MED may be immune to these resistance mechanisms. Furthermore, host-choice experiments indicated that there are likely different phytochemical blends in R1, R2 and R3 that repel and/or attract MEAM1, MED and NW1. The different responses of the three *B. tabaci* species to the three R lines reflect the divergence of these members of the *B. tabaci* species complex and stress the importance of considering the members of the *B. tabaci* species complex in a region prior to deployment of whitefly-resistance genotypes. It also should be noted that the MEAM1, MED and NW1 populations used in this study might reflect a subset of possible interactions, as many *B. tabaci* harbor different endosymbiont complements, which might influence their ability to interact with the four genotypes of alfalfa used in this study.

Based on the extreme repellence of NW1 to all four alfalfa lines assessed, alfalfa is an unlikely host in the field setting. These observations are well correlated with the lack of reports of whitefly-induced reductions in alfalfa quality or productivity prior to the MEAM1 invasion^74^. If alfalfa is chosen as a host, NW1 can oviposit and nymphs can develop on susceptible alfalfa genotypes; for this reason, alfalfa could serve as a bridging host to more NW1-susceptible plant species. While NW1 populations are once again being detected in the US^23^, its restricted host range relative to MEAM1 or MED^17,72^ means NW1 movement from alfalfa to adjacent crops may not be a significant threat to agriculture.

The discovery of differential resistance responses of three *B. tabaci* species to alfalfa’s whitefly-resistant lines was surprising and distinct from the whitefly-resistance mechanisms of cassava and tomato. For example, nymph mortality, lower fecundity, and repellence are all associated with the multigenic whitefly resistance in the cassava genotype ECU72^47–49^. However, ECU72 confers a broad-based resistance to seven whitefly species from four genera including: *Aleuotrachelus socialis*, *B. tabaci* SubSaharan African 1 (SSA1), *B. tabaci* SSA2, *B. tabaci* SSA3, *Bemisia tuberculata*, *Aleurothrixus aepim*, and *Trialeurodes variabilis*^47,75–78^. Furthermore, the apoplastic resistance to whiteflies expressed in *Mi1.*2 tomatoes confers similar levels of resistance to both MEAM1 and MED^37^. To date, the whitefly species specificity of *Brassica oleraceae*’s phloem-mediated antibiosis to the cabbage whitefly (*Aleyrodes proletella*) has not yet been explored^46^.

As alfalfa lines are genetically diverse populations of plants, which are designed to be resilient to changes in biotic stresses, deploying alfalfa’s multigenic resistance to *B. tabaci* is feasible and desirable. Considering the differences in resistance phenotypes of our R1, R2, and R3 lines, it might be beneficial to combine R1’s potent antibiosis that causes MEAM1 nymph mortality with R3’s antixenosis that repels both MEAM1 and MED and R3’s antibiosis that shortens the lifespans of both MEAM1 and MED. For deployment in the fields of California, the R1-R3 line would also need to express resistance to other pests and pathogens that limit alfalfa production. These traits were incorporated (but are not fixed) in the UC-2845 population (Fig. 1), from which R1 and R2 are derived; to date, we have not evaluated if these traits are present in the whitefly-resistant lines that we have studied. It is also noteworthy that Teuber et al.^79^ developed a whitefly-resistant alfalfa cultivar (UC-Impalo-WF) that was selected for high seed yields and adaptation to low desert growth conditions. It has varying degrees of resistance to multiple pathogens/pests that were also selected for the development of the UC-2845 population. In the future, it would be of interest to compare the mechanisms of resistance of UC-Impalo-WF to R1, R2 and R3 and assess the performance on MEAM1, MED and NW1 on UC-Impalo-WF individuals.

It has been proposed that 98–99% of nymphs must perish to effectively manage whitefly populations in the field^16^. Our simulation model indicates that deployment of the high levels of nymph mortality displayed in the R1, R2 and R3 lines would have a profound impact on whitefly population expansion. Our model predicts that whitefly-susceptible alfalfa support whitefly populations that are 11 million-fold higher than whitefly-resistant alfalfa. In addition, we show that the presence of other antibiotic and antixenotic traits, in addition to nymph mortality, influences the potency of the whitefly-resistance mechanisms of alfalfa. Our models predict that in the R1 and R3 backgrounds, nymph mortality can be reduced to 95% or 90% without significant population expansion, respectively. In addition, significant delays in nymph development may enhance biocontrol, as longer windows of opportunity for predators and parasitoid wasps to identify whitefly nymphs would be provided^80,81^. Our simulation model does not incorporate the strong repellence mechanisms active in R1, R2 and R3 nor losses that occur naturally in the field (i.e., nymph dislodgment and nymph mortality due to predators and parasitoids)^16^. These additional events should enhance the effectiveness of R1, R2 and R3 whitefly resistance in the field.

Finally, given the unique genetic identify of each resistant plant in the UC-2845 and UC-2933 populations, we have a rich resource to identify other resistance traits. One untapped resource is the alfalfa lines that are moderately resistant to MEAM1. This premise is supported by the fact that the UC2845-050 line, which caused 85% MEAM1 nymph mortality, caused 100% NW1 nymph mortality. Future studies should explore the nuanced differences between highly-resistant and moderately-resistant alfalfa and identify the metabolites that underlie alfalfa’s potent whitefly resistance. Our current transcriptome and metabolome studies should reveal the molecular, biochemical and cellular mechanisms that control this novel nymph-based whitefly resistance mechanism.

## Methods

### Host plants and *B. tabaci* colony maintenance

The *Bemisia tabaci* MEAM1 colony was maintained on *Brassica juncea* var ‘Florida Broad Leaf’ (W. Atlee Burpee & Co.) grown in UC Soil mix 3 at 27 °C, 55% relative humidity and 16-h light:8-h dark cycle (300 µmol m^− 1^ sec^− 1^). The MEAM1 colony has been maintained in isolated at UCR since 1998^21,82^; this line of MEAM1 contains only the primary endosymbiont Portiera (*Candidatus Portiera aleyrodidarum*)^83^ and the secondary endosymbiont *Hamiltonella defensa*^84^. The *B. tabaci* MED colony was initiated with adults provided by Jesús Navas-Castillo (University of Málaga). The MED colony was maintained under similar conditions with quarantine protocols in UCR’s Insectary and Quarantine facility (IQF). The *B. tabaci* NW1 colony was initiated with whiteflies provided by James Ng (UCR). The NW1 colony was maintained on *Phaseolus vulgaris* var ‘Fordhook’ (W. Atlee Burpee & Co.) in a separate room in IQF with the same conditions described above.

### Generation and propagation of whitefly-resistant and -susceptible alfalfa lines

Teuber et al. identified MEAM1 resistance in the alfalfa (*Medicago sativa* L.) UC-356 germplasm pool^60^. UC-356 was used to create the three alfalfa populations with varying levels of MEAM1 resistance/susceptibility that were used in this study (Supplementary Figure S1). Plants from UC-356 were subjected to one cycle of selection for increased whitefly-susceptibility to generate the UC-1872 population (Supplementary Figure S1). A multi-year breeding project was initiated with 71 whitefly-resistant individuals from UC-356^60^. Four cycles of selection for whitefly resistance created the UC-2458 population; while individuals from the UC-2458 population were used in Jiang et al. (2003), the name of this population was not previously reported. UC-2458 was the progenitor of both resistant populations (UC-2845 and UC-2933) used in this study (Supplementary Figure S1).

The UC-2933 germplasm was created from four highly resistant individuals (clones 3, 10, 27 and 37) characterized in Jiang et al. (2003)^59^. These individuals were used to create half-sib families (UC-2527-26, UC-2458-34, UC-2527-60, and UC-2458-177, respectively) (Supplementary Figure S1). These half-sib families were used to create the UC-2933 germplasm. To generate UC-2845, UC-2458 germplasm was subjected to three cycles of selection resistance to MEAM1 and the spotted alfalfa aphid (*Therioaphis maculata*), pea aphid (*Acyrthosiphon pisum)*, and bluegreen aphid (*Acyrthosiphon kondoi*), *Phytophthora megasperma* f. *medicaginis*,* Fusarium oxysporum* f. sp. *medicaginis*, *Colletotrichum trifolii*, and northern and southern root-knot nematodes (*Melodigyne* spp) (Supplementary Figure S1).

Cuttings from 84 individuals from the UC-1872, UC-2845 or UC-2933 populations were collected from field-grown alfalfa (El Centro, CA) or from clones (UC Davis) to establish parent plants. Stem segments (6-cm) were clonally propagated to establish parent plants. Clones of parent plants were made for phenotypic screens and bioassays. The details of alfalfa asexual propagation are provided in *Supplementary Methods*.

### Whitefly resistance/susceptibility bioassays

To screen a large number of alfalfa lines for MEAM1 resistance, we adapted the methods of Jiang et al.^59^ to obtain a snapshot of nymph development at the time a pseudopupa, exuvium, or adult appeared on a MEAM1-susceptible genotype (CUF-101 or UC-2845-043) (*Supplementary Methods* for details). In each screen, alfalfa lines (*n* = 4–9) with unknown resistance/susceptible status were evaluated. To facilitate the large number of simultaneous infestations, we established sex-specific holding plants (600 whiteflies/plant).

Alfalfa plants (five trifoliate leaves) were moved into Bugdorms in a greenhouse with day-time temperatures ~ 23^o^C and natural light. On the day of an infestation, two leaves per plant were enclosed in cages with six male and six female whiteflies (Supplementary Figure S5). After 48 h, cages were removed, the number of viable adults/per cage recorded, and infested leaves were marked with a jewelry tag. A random block design was used for all infestations. Infestations were terminated when a pseudopupa, exuvium, or adult was observed on the susceptible line. At this time, infested leaves were excised; the abaxial and adaxial sides of each leaflet were photographed using the Nikon D5000 at UCR’s Microscopy and Imaging Core. The number of first, second, third, and fourth instars and exuvia were determined. The percentage of insects in each developmental stage was determined by the number of insects in each instar divided by the total number of instars/exuvia.

For each line, the percentage of nymphs in their first instar was determined; each screen was analyzed using a Kruskal-Wallis One-Way ANOVA and subsequent Dunn’s multiple comparison tests (Supplementary Table S1).

Data from one representative screen is shown in Supplementary Figure S2. The percentage of insects in their first instar was used to define five classes of resistance/susceptibility. The significance of the mean percent of insects in their first instar for each line (*n* = 5–10) was assessed using a Kruskal-Wallis One-Way ANOVA. Data was arcsin transformed. Experiments with a *p* < 0.05 indicated at least one line in the screen displayed a resistant phenotype. Resistant lines were confirmed with Dunn’s multiple comparison tests against the known susceptible line. The complete data set and analysis is in Supplementary Table S1. The susceptible genotype 2845-043 (S1) and three highly resistant genotypes 2845-092 (R1), 2845 − 100 (R2) and 2933-022 (R3) were selected for further analysis. Leaf and stem morphologies for the S1, R2, and R3 lines were the same, while the leaves of R1 were narrower (Supplementary Figure S6).

### MEAM1, MED1 and NW1 nymph development on resistant and susceptible lines

Development of MEAM1 and MED1 nymphs on R1, R2, R3 and S1 (susceptible control) and NW1 nymphs on CUF-101 (susceptible control), R2, R3, and two lines moderately resistant to MEAM1 (UC-2845-050 and UC-2933-010) was assessed using the infestation protocols described above for the high-throughput screen. The percentage of first-instars for MEAM1 and MED1, and all instars from NW1 experiments were analyzed by performing a Kruskal-Wallis One-Way ANOVA at the 0.05 confidence interval. Multiple comparisons were performed with a Dunn’s test with the known susceptible genotype serving as the control. Percentage of instars were arcsin transformed before statistical analysis.

### Oviposition assays

For each alfalfa line (S1, R1, R2, and R3), two young trifoliate leaves from five 5-in tall plants were enclosed in cages and infested with two male and two female *B. tabaci* (MEAM1, MED or NW1). Each line was screened twice (*n* = 20). After 48 h, adult viability was determined, leaves were excised and the number of eggs on the abaxial and adaxial leaf surfaces were determined. The eggs from each replicate of a line were summed and divided by the sample size to determine the mean oviposition rate. Egg oviposition was analyzed using a Kruskal-Wallis H-test. Significantly different samples were determined with a Dunn’s multiple comparison test.

### Adult-choice experiments

Two-way choice “cages” design and details of the choice experiments are described in *Supplementary Methods*. Thirty whiteflies (MEAM1, MED or NW1) were released into a cage with two alfalfa plants (Supplementary Figure S7). Each experiment used S1 and one whitefly-resistant line (R1, R2, or R3); there were five biological replicate experiments. At 8, 24, 48, and 72 hpi (hours post-infestation), the number of whiteflies residing on the adaxial and abaxial side of leaves was determined. A flashlight was used to illuminate the leaf from below and the shadows of the whiteflies residing on each leaflet were counted when whiteflies were not directly visible. Whiteflies that died or were not found on a plant were called as no-choice decisions. The number of whiteflies on each line or making no choice were divided by the total number of whiteflies in the cage to determine the proportion of whiteflies choosing the S or R plants. Adult-choice experiments were analyzed using a two-way RM ANOVA with a Geisser-Greenhouse correction on arcsin-transformed proportions at each time point. Significantly different samples were determined using a Tukey’s multiple correction test with individual variances calculated for each comparison. Each experiment was conducted at 26 °C and 200–300 µmol m^− 1^ sec^− 1^ light with a 12-h day.

### Longevity studies

Five pairs of newly emerged MEAM1 or MED adults (1:1 sex ratio) were added to a caged trifoliate leaf from each alfalfa plant (with 8–10 leaves). See *Supplementary Methods* for details. The number of alive and dead whiteflies per cage was determined in 24-h intervals for 24 d. Five replicates (*n* = 5) were completed for each line. Survival curves were compared using a Mantel-Cox test at the 0.05 interval. Significantly different samples were determined by comparing survival curves between two genotypes in an experiment.

### Simulation model

Our model is based on the population growth model of van Giessen et al.^64^. We used one day as the time step and the population was divided into cohorts of individuals of the same age in days. The individuals within a cohort develop with identical and fixed parameter values. Six parameters were used in our simulation model.

(1) Nymph Mortality Rate (NMR): the percentage of nymphs that do not complete their development and reach adulthood. According to Jiang et al. (2003)^59^, if a nymph reaches its 2nd instar on an alfalfa whitefly-resistant line, the insect will survive to adulthood. Therefore, we set the NMR for R1, R2 and R3 based on the percentage of nymphs that do not progress beyond their 1st instar.

(2) Duration of Nymphal Stage (DN): the number of days from egg deposition to adult emergence on a susceptible host is based on Yee and Toscano (1996)^1^, DN = 23 for MEAM1 on CUF-101.

(3) Maturation Period (MP): the number of days between adult emergence and egg deposition. While there is variation in the MEAM1 MP values in the literature, most indicate MP is less *≤* 1 day^85–89^. We used MP = 1 in our simulation model.

(4) Adult Mortality Rate (AMR): the percentage of adults that die after a certain number of days. These data are derived from our longevity studies.

(5) Female Percentage (FP): the percentage of females among adults in a cohort. Most MEAM1 populations display some degree of female bias, which can be temperature dependent^65^. However, there are studies that indicate the MEAM1 male: female ratio = 1^90^. We used two FP values in our simulation model (FP = 55% and FP = 65%).

(6) Oviposition Rate (OR): the number of eggs a female deposits per day. Although oviposition is age dependent, we assumed that all females have a constant OR in the model. OR values were based on our oviposition studies.

## Electronic supplementary material

Below is the link to the electronic supplementary material.

Supplementary Material 1

Supplementary Material 2

Supplementary Material 3

Supplementary Material 4

## Data Availability

All the data and materials integral to this study are available within the article and the Supporting Information.
