# Supplementary material for: Medicago sativa’s antixenotic and antibiotic resistance mechanisms differentially impact three members of the Bemisia tabaci species complex
Source: Sci Rep. 2025 Oct 16;15:36134. doi: 10.1038/s41598-025-01426-z (PMC12533143; doi:10.1038/s41598-025-01426-z)
Supplement: Supplementary file 3 — Supplementary Material 3 [file 41598_2025_1426_MOESM3_ESM.pdf]

Thomas P., Benabderrahim M.A., Li J., Jiu M., Wang L., Holzer F., Teuber, L. and Walling L.L. 2025. *Medicago sativa*'s antixenotic and antibiotic resistance mechanisms differentially impact three members of the *Bemisia tabaci* species complex

Supplemental Methods

Asexual propagation of alfalfa lines

Cuttings from 84 individuals from the UC-1872, UC-2845 or UC-2933 populations were collected from field-grown alfalfa (El Centro, CA) or from clones (UC Davis) to establish parent plants. Stem segments (6-cm in length) were clonally propagated in UC soil mix 3 by dipping the distal end of the cutting into Clonex gel rooting media (Growth Technology Ltd) and in Spinosad insecticide (Tractor Supply Co., Brentwood, TN) to eliminate herbivores accidently brought in from the field. Stem cuttings were placed in soil in a 72-well inserts, with three stem segments per well. Stem segments were housed under a humidity dome (Hydrofarm, Petaluma, CA) and misted daily. Dome vents were opened after clones established roots (approximately 10 – 14 d) and removed after 21 d. Stem segments with established root systems were transferred to 5-inch tall rectangular pots and were grown in a growth room at 27°C, 35-50% relative humidity with a 12-h day/12-h night cycle (200 – 300 µmol m^{-1} sec^{-1}). Established plants were transferred to 1-gallon pots and parent plants of each genotype were maintained in a greenhouse or growth room with monthly fertilization.

Whitefly resistance/susceptibility bioassays

Alfalfa lines were screened for whitefly resistance/susceptibility using a method adapted from Jiang *et al.* (2003). To screen a large number of alfalfa lines, we obtained a snapshot of whitefly nymph development at the time 4th instars (pseudopupae), exuvia, or an adult appeared on a MEAM1-susceptible genotype (Figure 2). CUF-101 (Jiang *et al.*, 2003) and UC-2845-043 were used as susceptible controls in early and later phenotypic screens, respectively. In each screen, alfalfa lines with unknown whitefly resistance/susceptible phenotypes were evaluated. Early screens assessed four lines with ten replicate plants. Statistical evaluation of the data indicated that phenotypes could be accurately assessed with five plants per line. Later screens assessed nine lines with five replicate plants.

To facilitate infestations, we established sex-specific holding plants (600 whiteflies/plant). Individual whiteflies were collected in 50-mm test tubes from MEAM1 colonies, capped with

corks and the sex of each whitefly was verified under a dissecting microscope. Male- and female-holding plants (*B. juncea*) were established in separate Bugdorms (MegaView Science Company) in the greenhouse; insects were used one to two days later.

Alfalfa plants (five trifoliate leaves) were moved into Bugdorms in a greenhouse with day-time temperatures ~23°C and natural light; screens were performed from March to October. On the day of an infestation, cages were placed on two young alfalfa trifoliate leaves per plant. Cages were adapted from a design of Jiang *et al.* (2003). Infestations were initiated by delivering six male and six female whiteflies into each insect cage. After 48 h, cages were removed, the number of viable adults/per cage recorded, and infested leaves were tagged with a jewelry tag. A random block design was used for all infestations. Infestations were terminated when a pseudopupa, exuvia, or adult was observed on the susceptible line. At this time, infested leaves were excised; the abaxial and adaxial sides of each leaflet were photographed using the Nikon D5000 at UCR's Center for Plant Cell Biology Microscopy and Imaging Core. The number of first, second, third, and fourth instars and exuvia were determined. The percentage of insects in each developmental stage was determined by the number of insects in each instar divided by the total number of instars/exuvia.

The percentage of insects in their first instar was used to define five classes of resistance/susceptibility. The significance of mean percent of insects in their first instar for each line (n=5-10) was assessed using a Kruskal-Wallis One-Way ANOVA. Data was arcsin transformed. Experiments with a $p \leq 0.05$ indicated at least one line in the screen displayed a resistant phenotype (Supplementary Table S1). Resistant lines were confirmed with Dunn's multiple comparison tests against the known susceptible line. The susceptible genotype 2845-043 (S1) and three highly resistant genotypes 2845-092 (R1), 2845-100 (R2) and 2933-022 (R3) were selected for further analysis.

Adult choice experiments

The two-way choice cage is diagrammed in Fig. 4a. These cages are a modification of the four-way choice cages described in Kenney *et al.* (2020). Hinged plastic boxes (140 mm x 168 mm x 76 mm) (mDesign) had two 2.5-cm diameter holes drilled at the base of the cage for insertion of alfalfa plants and a 7.5-cm x 5-cm opening at the back of the box was covered in thrips-proof mesh to allow for air flow. The boxes had a central hole (2.5-cm diameter) to which the cap of a 50-ml tube with a central 2.5-cm hole was glued. The cap allowed attachment of the whitefly collection tube, which was a 50-ml centrifuge tube that was truncated at the 40-ml line and sealed with thrips-proof mesh. The collection tube was

screwed into the cap to initiate the choice experiment. Cages were mounted on a ring stand using a clamp 30 cm from the tabletop. Each experiment used S1 and one whitefly-resistant line (R1, R2, or R3) with 5 to 8 leaves with five biological replicate experiments. Plants were introduced to the cage by inserting a stem with three trifoliate leaves into the cage and sealing the hole with 3.2-cm^3 of insulation foam. Each experiment used S1 and one whitefly-resistant line (R1, R2, or R3) with 5 to 8 leaves with five biological replicate experiments. Plants were introduced to the cage by inserting a stem with three trifoliate leaves into the cage and sealing the hole insulation foam. Prior to the addition of whiteflies, the inside of cages was wiped with a water-dampened Kimwipe to minimize the static electricity, which negatively impacts whitefly release. Thirty whiteflies were collected from the MEAM1, MED or NW1 colonies and held for 15 min at room temperature or 4°C to ensure the whiteflies were at the bottom of the collection tube. The collection tube was screwed into the cage and the tube was gently tapped to ensure all whiteflies were released. Cages were surrounded with white cardstock to minimize external stimuli. Choice cages were left undisturbed except for daily watering and data collection. Monitoring of plant choice and statistical analyses are described in *Materials and Methods*.

Longevity studies

Whitefly cages were created using 236-ml plastic containers with a 2.5-cm hole cut in the bottom of the container (for insertion of the plant), two 3-cm holes sealed with thrips-proof mesh on opposite sides for ventilation, and a 0.5-cm hole to deliver whiteflies. Cages were mounted on sticks using heavy wire to prevent bending or damage to the leaf petiole. A trifoliate leaf from each alfalfa plant (with 8 – 10 leaves) was caged and sealed with 3.2 cm^3 of insulation foam and infested with whiteflies. The number of alive and dead whiteflies per cage was determined in 24-h intervals for 24 d. Whiteflies were transferred to a clean leaf on the same plant every 7 d or when the infested leaf was showing signs of damage. Five replicates (n=5) were completed for each line. Statistical analysis of data are described in *Materials and Methods*.
