# Supplementary material for: Medicago sativa’s antixenotic and antibiotic resistance mechanisms differentially impact three members of the Bemisia tabaci species complex
Source: Sci Rep. 2025 Oct 16;15:36134. doi: 10.1038/s41598-025-01426-z (PMC12533143; doi:10.1038/s41598-025-01426-z)
Supplement: Supplementary file 4 — Supplementary Material 4 [file 41598_2025_1426_MOESM4_ESM.pdf]

Thomas P., Benabderrahim M.A., Li J., Jiu M., Wang L., Holzer F., Teuber, L. and Walling L.L. 2025. *Medicago sativa*'s antixenotic and antibiotic resistance mechanisms differentially impact three members of the *Bemisia tabaci* species complex.

Supplementary Figures

Supplementary Fig S1. Breeding Strategy for whitefly-resistant and -susceptible populations.

Supplementary Fig S2. The percentage of first-instar nymphs on six alfalfa lines in a representative whitefly-resistance screen.

Supplementary Figure S3. Distribution of plants from the UC1872, UC2845, and UC2933 populations in whitefly-susceptible vs -resistant phenotype classes.

Supplementary Figure. S4. Simulation model for MEAM1 growth on R1, R2, R3 and S1 alfalfa using a FP of 65%.

Supplementary Fig. S5. Phenotypic screen to identify whitefly-resistant and -susceptible alfalfa individuals.

Supplementary Figure S6. Leaf morphology of three resistant and a susceptible line.

Supplementary Figure S7. Free-choice experiment diagram.

Supplemental Figure S1

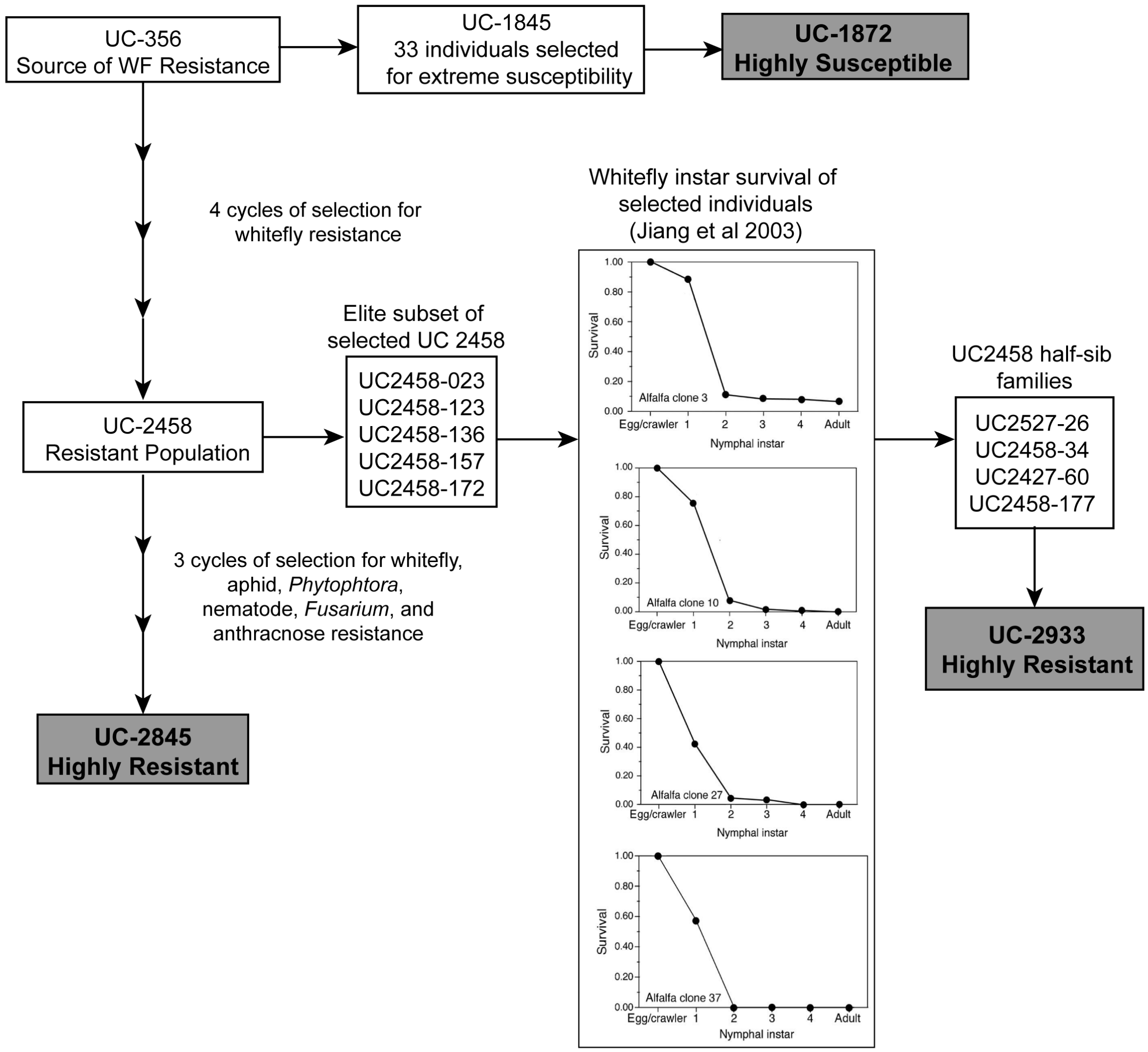

Fig. S1. Breeding strategy for whitefly-resistant and -susceptible populations.

Alfalfa whitefly-resistant germplasm was developed from UC-356 using a shuttle breeding scheme as described by Teuber et al (1997). This germplasm was used to create a resistant population (UC-2458), which was subsequently used to create two elite populations of whitefly-resistant alfalfa (UC-2933 and UC-2845). A highly susceptible population (UC-1872) was also made from the U-C356 germplasm by selecting for 33 highly susceptible lines. Details of the breeding scheme are in *Materials & Methods.*

Supplementary Figure S2

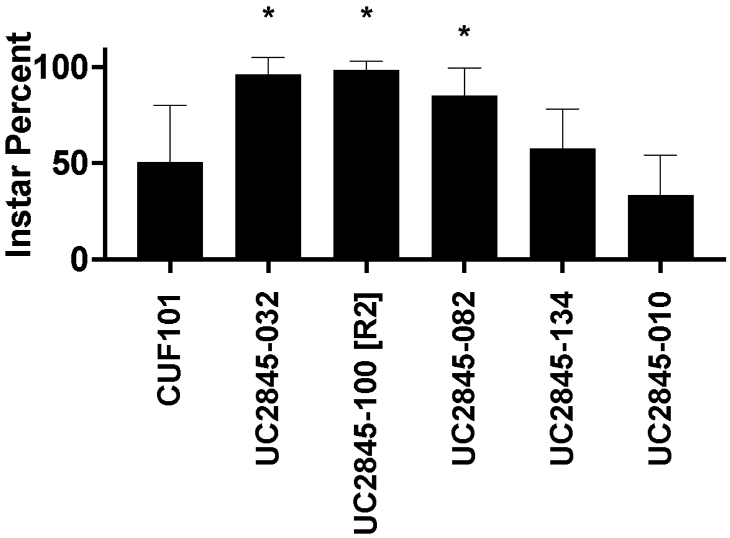

Fig. S2. The percentage of first-instar nymphs on six alfalfa lines in a representative whitefly-resistance screen.

The number of nymphs in each instar was determined in five genotypes from UC2845 population (n=12) and CUF-101 (the susceptible control) when 4th instars were detected on CUF-101. The percentage of insects in their first instar was determined and compared using a Kruskal-Wallis One-Way ANOVA after arcsin square root transformation of each mean. The experiment had significant differences in first-instar mortality ($p < 0.0001$). Resistant genotypes were confirmed by conducting a Dunn's multiple comparisons test against CUF-101. Resistant genotypes that passed the Dunn's multiple comparison threshold ($p<0.05$) are indicated with an asterisk. UC-2845-100 (R2) and UC2845-032 were classified as highly resistant, UC2845-082 was moderately resistant, and UC-2845-134 was moderately susceptible relative to susceptible CUF-101 and UC2845-010 plants ($p < 0.01$).

Supplementary Figure S3

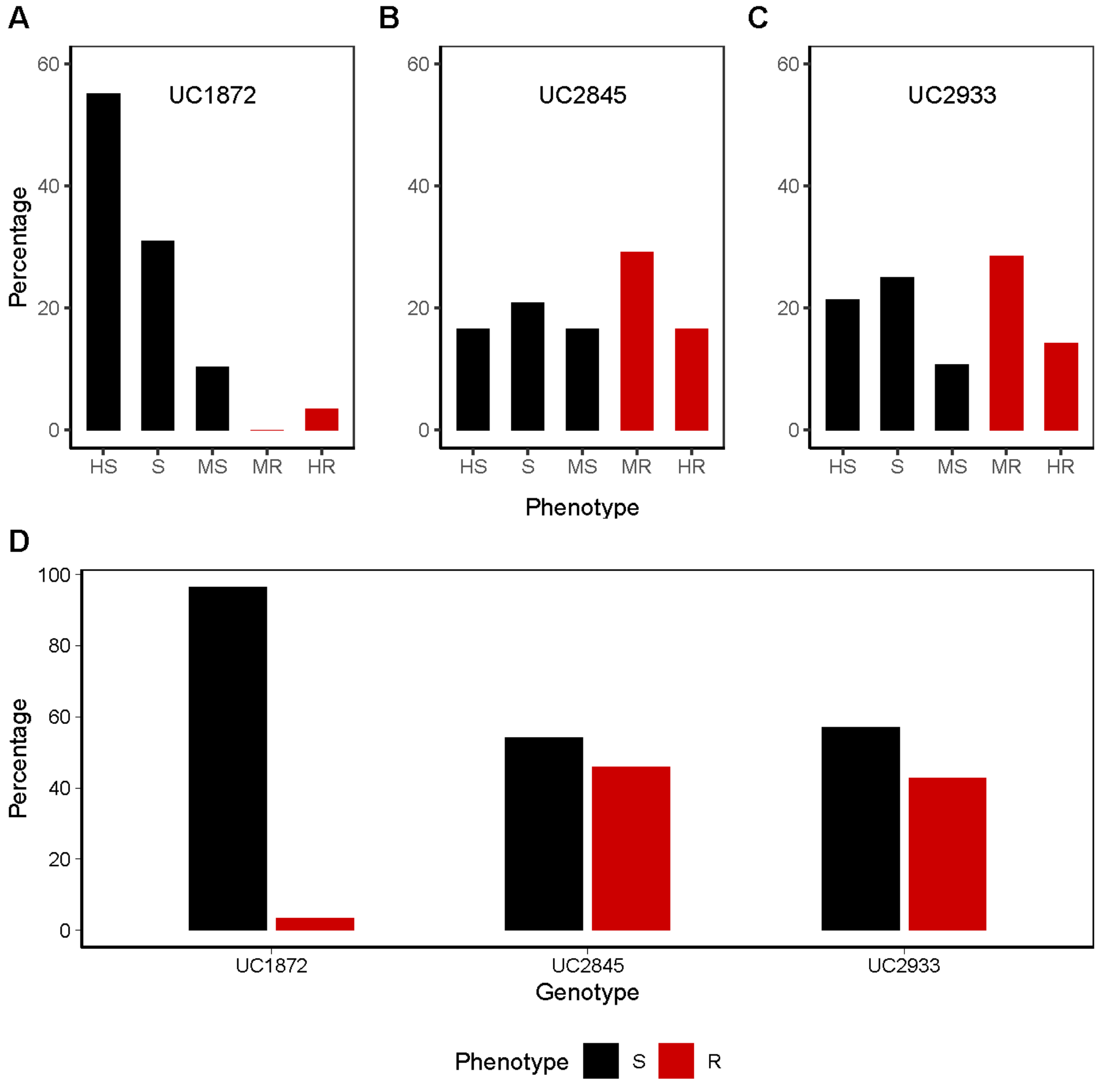

Fig. S3. Distribution of plants from the UC1872, UC2845, and UC2933 populations in whitefly-susceptible vs -resistant phenotype classes.

a-c, The percentage of plants ranked as highly (HR), moderately resistant (R class), moderately susceptible (MS), susceptible (S) and highly susceptible (HS class) is displayed for the UC1872, UC2845, and UC2933 populations. **d,** The percentage of plants ranked as HR or R (red) vs MS, S or HS (black) are shown.

Supplementary Figure S4

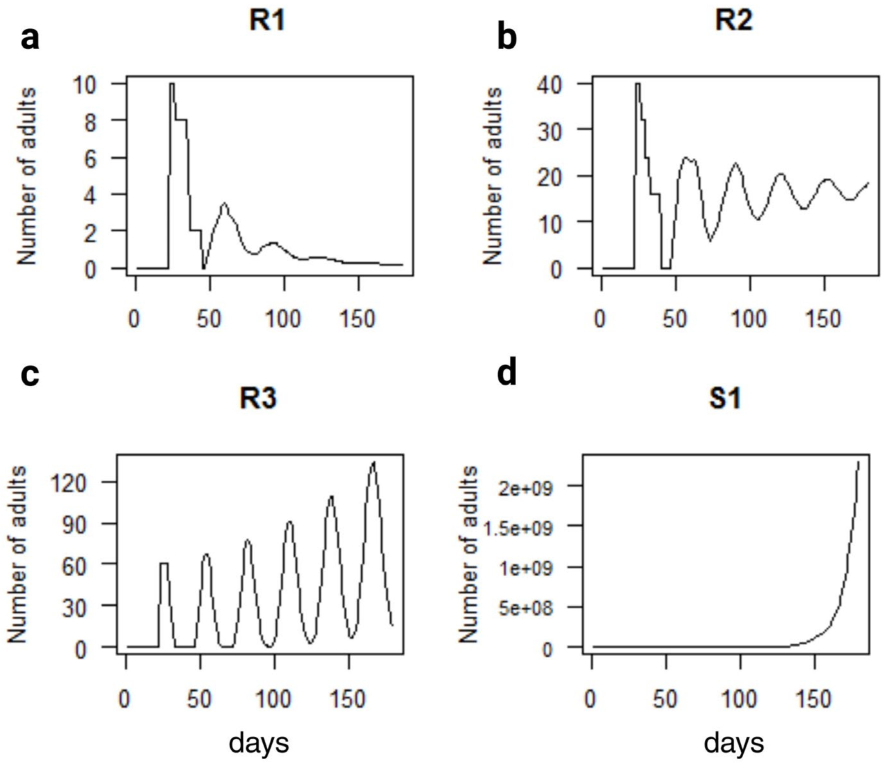

Fig. S4. Simulation model for MEAM1 growth on R1, R2, R3 and S1 alfalfa using a FP of 65%.

The parameters used in this model are the same as in Figure 6, however a FP = 65% was used. Population growth on R1 (**a**), R2 (**b**), R3 (**c**) and S1 (**d**).

.

Supplementary Figure S5

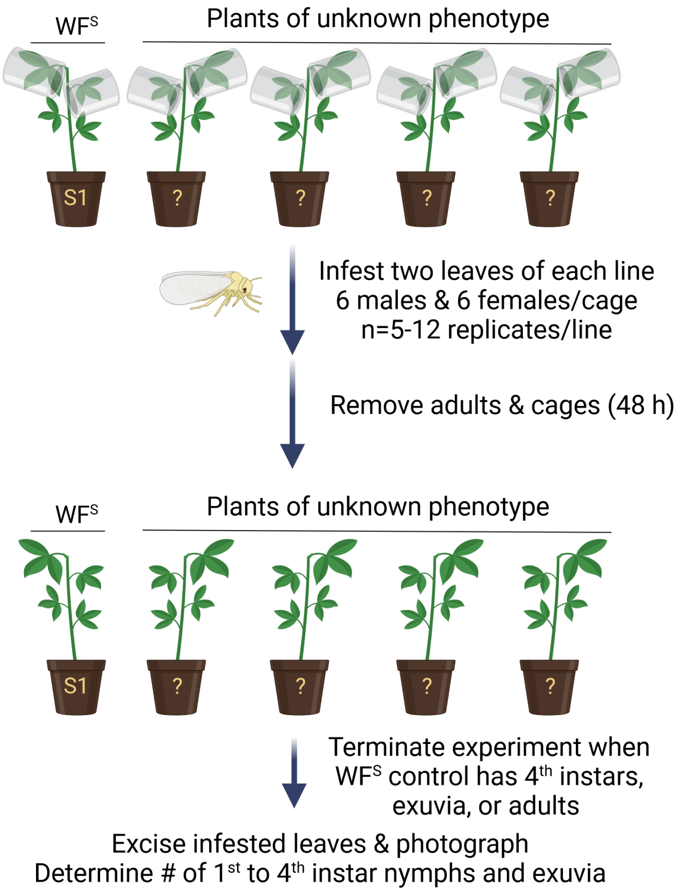

Fig. S5. Phenotypic screen to identify whitefly-resistant and -susceptible alfalfa individuals

Two leaves of each plant were infested with six male and six female *B. tabaci* MEAM1 with 5 to 12 replicates per line. One whitefly-susceptible (WF^S) genotype (CUF101 or UC-2845-043) was included in each screen as a positive control. After 48 h, adults and cages were removed. The experiment was terminated with WF^S plants had late-stage 4th instars, exuvia or adults. Infested leaves were excised and the numbers of insects at each nymphal stage and exuvia were counted.

Supplementary Figure S6

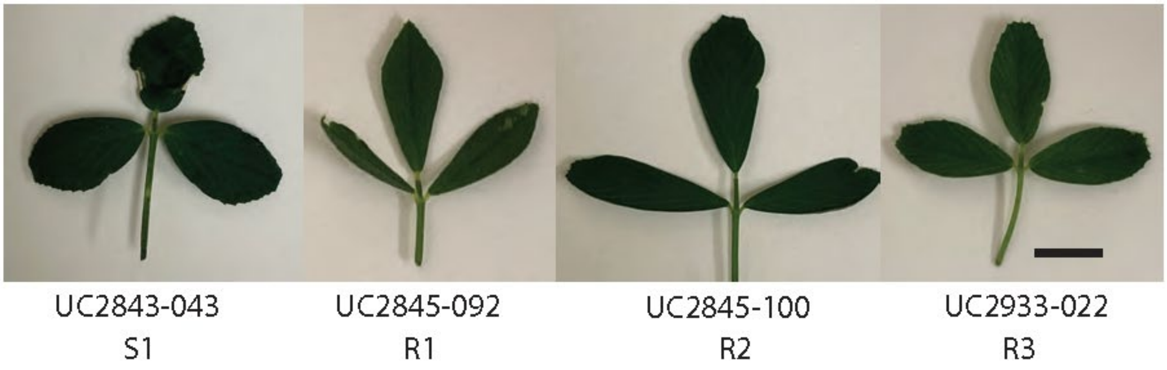

Fig. S6. Leaf morphology of three resistant and a susceptible line.

Representative leaves from the R1 (UC2845-092), R2 (UC2845-100), R3 (UC2933-022) and S1 (UC2845-043) lines are displayed.

Supplementary Figure S7

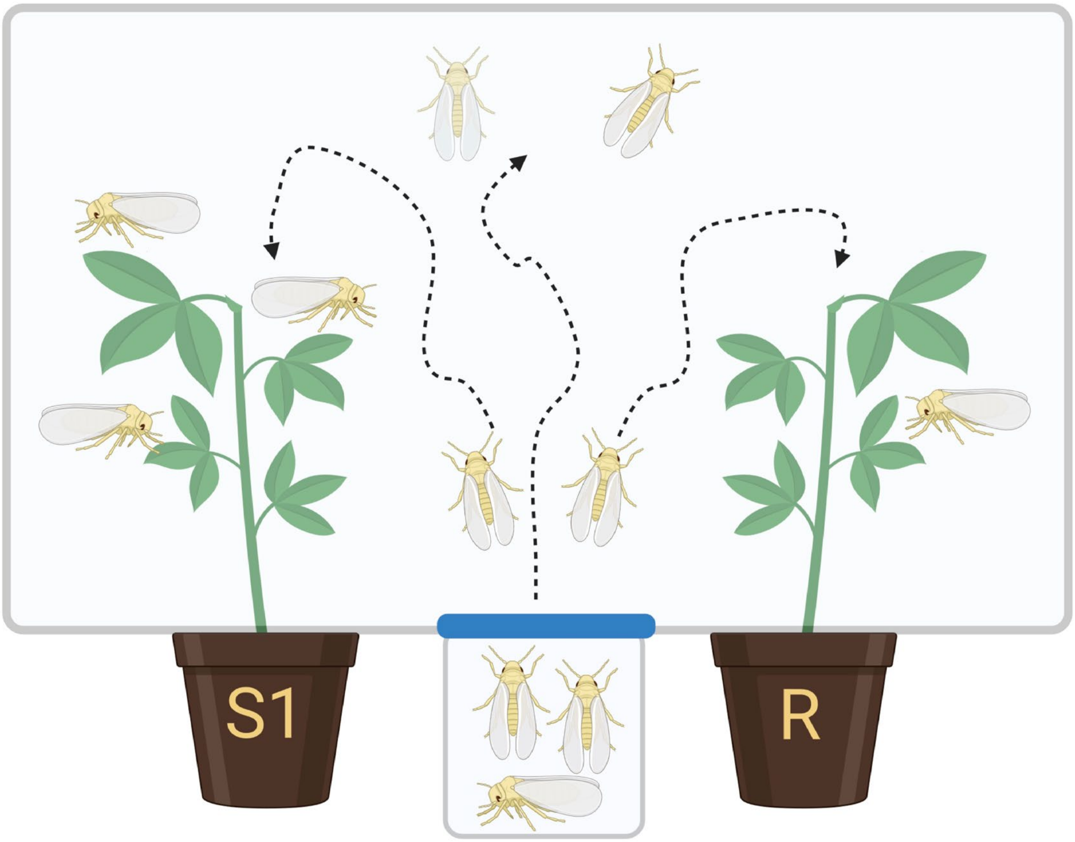

Fig. S7. Two-way choice studies.

Whiteflies were released into a two-way choice cage. Number of adults on the S1or a resistant line (R1, R2, or R3) were determined. Whiteflies that died or were located on the cage rather than on a plant were considered no-choice actions.
